# Characteristics of pediatric interventional drug trials registered between 2015 and 2024 on ClinicalTrials.gov

**DOI:** 10.3389/fped.2025.1695990

**Published:** 2025-12-10

**Authors:** Yinghong Zhou, Zhaoxin Liu, Ji Xu, Ying Zhang, Haiqi Shen, Siyu Jiang, Yunyun Shi, Yinghua Lv, Jihan Huang

**Affiliations:** 1 Science and Information Center, Shanghai University of Traditional Chinese Medicine, Shanghai, China; 2 Center for Drug Clinical Research, Institute of Interdisciplinary Integrative Medicine Research, Shanghai University of Traditional Chinese Medicine, Shanghai, China

**Keywords:** pediatric, clinical trial, interventional drug trial, dosage form, route of administration

## Abstract

**Aim:**

This study comprehensively analyzed the characteristics and development trends of pediatric interventional drug clinical trials registered between 2015 and 2024, with a focus on pharmaceutical characteristics such as dosage form and administration route.

**Methods:**

The ClinicalTrials.gov database was searched to identify trials involving pediatric participants. Interventional trials first posted between 2015 and 2024 were included if they used drugs as interventions and had treatment as the primary purpose.

**Results:**

A total of 2,928 pediatric-only clinical trials were included, with annual registration numbers ranging from 257 to 339, exhibiting a declining trend. Among these, 1,975 (67.5%) enrolled no more than 100 participants, and 153 (5.2%) were restricted to either males or females, with this gender-specific enrollment correlated with disease epidemiology. Mental, behavioral, or neurodevelopmental disorders (319, 10.9%) represented the most studied therapy area, maintaining high proportions consistently across Phases 1–3. The most common dosage forms were liquid (53.3%) and solid (24.0%), and the most frequently reported administration routes were enteral (39.0%) and parenteral (35.6%). Drug formulations and administration routes varied by age group: the use of solid formulations (from 12.1% to 29.1%) and enteral administration (from 22.9% to 42.0%) increased with age, while the use of liquid formulation and parenteral administration declined.

**Conclusion:**

In the last ten years, registered pediatric drug trials have either stayed stable or slightly decreased, often featuring small sample sizes and rarely using age-specific formulations. National regulatory bodies should boost policy support to encourage pediatric drug research and improve trial design quality.

## 1 Introduction

The registration of clinical trials is a fundamental requirement of medical research ethics, representing both an obligation and a moral responsibility for investigators. This practice facilitates the traceability of clinical trial outcomes and enhances the public availability and transparency of trial information (1). ClinicalTrials.gov is an online platform and database that provides detailed information on clinical research studies and their outcomes. The aim of this platform is to make information on such studies accessible to the public, researchers, and health-care professionals (2). Clinical trial registration improves the traceability of results and strengthens the openness of trial information, thereby enhancing the scientific validity and transparency of the research (3). The field of pediatrics is characterized by ongoing growth and development, which leads to physiological, pathophysiological, pharmacokinetic, and pharmacodynamic differences not only between pediatric and adult populations but also among various stages of childhood. Pediatric clinical trials are essential to ensuring that children receive safe, effective, and developmentally appropriate treatments (4).

Studies have explored the basic characteristics of pediatric clinical trials registered on ClinicalTrials.gov. For instance, Pasquali et al. (5) analyzed all interventional trials conducted between July 2005 and September 2010, examining their characteristics, treatment areas, locations, and sponsors. Zhong et al. (6) conducted a systematic review of 53,060 pediatric clinical studies published from January 2008 to December 2019, including observational clinical trials. Other studies have analyzed trials within specific pediatric disease areas. For example, Wang et al. (7) explored childhood obesity trials, Awerbach et al. (8) analyzed pediatric pulmonary hypertension trials, Desselas et al. (9) evaluated randomized controlled drug-vs.-placebo trials in newborns, and Hill et al. (10) investigated pediatric cardiovascular disease trials.

Although some studies have assessed pediatric trials focusing on specific diseases or regions, others have investigated global pediatric drug development. Nonetheless, the most recent comprehensive data specifically analyzed clinical trials conducted during the period from 2008 to 2019 (6). Few have comprehensively analyzed the characteristics of global pediatric interventional drug trials conducted between 2015 and 2024. During this decade, pediatric drug development has faced novel opportunities and challenges, including modifications in pediatric regulations (11, 12), COVID-19 disruption, emerging therapeutic classes such as biologics (13). An update spanning a decade is particularly justified to elucidate the characteristics of pediatric drug development within the contemporary research and development ecosystem. This includes an analysis of trends, distribution across various disease areas, and the selection of dosage forms and routes of administration. This gap is particularly evident in analyses of trends in the registration of pediatric drugs and the evaluation of disease areas, dosage forms, and administration routes over the past decade.

In the present study, we retrieved data on pediatric interventional drug trials from the ClinicalTrials.gov database for the period 2015 to 2024 and performed a multidimensional classification with descriptive statistical analysis. A comprehensive understanding of the landscape of pediatric drug clinical trials over the past decade may enhance the engagement of stakeholders in pediatric drug development and facilitate the formulation of new regulatory policies.

## 2 Methods

### 2.1 Data source and search strategy

All data used in this study were obtained from ClinicalTrials.gov (https://clinicaltrials.gov/), a publicly accessible registry and results database of privately and publicly funded clinical studies conducted worldwide. Search was conducted by using the following strategy: age [Child (birth—17)], study type (interventional), and date of first posting (January 1, 2015 to December 31, 2024). There were no more limitations on other search items. Data were directly exported from ClinicalTrials.gov in the CSV format and imported into Microsoft Excel for cleaning.

### 2.2 Study selection

This study sought to examine the current state of pediatric drug clinical trials that utilized pharmacological interventions for therapeutic research purposes. To accomplish this objective, we implemented the following procedure to screen the trials: (1) Retained trials categorized as “child” according to age of eligibility, while excluding those labeled as “child, adult” or “child, adult, older-adult”; (2) Excluded studies involving non-pharmacological interventions, such as behavioral, device, dietary supplement, procedural, or radiation interventions; (3) Retained studies with treatment as the primary purpose, as indicated in the primary purpose field within the study design, and excluded those with primary purposes such as basic science, diagnostic, prevention, or screening; (4) Further excluded trials related to animals or fetuses based on their titles, abstracts, and protocols. Two reviewers assessed all identified trials to determine their eligibility for inclusion. In instances of disagreement, a third reviewer was consulted to facilitate a resolution.

### 2.3 Data extraction

Data extracted included National Clinical Trial number, study title, results, conditions, interventions, sex, age, healthy volunteers, phases, enrollment, study type (allocation, intervention model, and masking), and first publication date. In addition to these variables, we manually labeled the following core variables: (1) therapy area, which was defined according to conditions, using the International Classification of Diseases, Eleventh Revision (ICD-11) code as a reference; (2) age subgroup, defined according to detailed age derived from protocol. Pediatric populations were classified into four age subgroups: newborns (birth to 1 month of age), infants (1 month to 2 years of age), children (2–12 years of age) and adolescents (12–17 years of age) according to the definition given by the International Council for Harmonization (ICH) and U.S. Food and Drug Administration (FDA); (3) dosage form, which was classified according to Dash and Limenh's research (14, 15); (4) route of administration. Data for the latter three variables were obtained from the clinical trial protocols. For ambiguous or missing data, further information was collected and verified by searching medical databases and search engines using the National Clinical Trial number as the keyword. If the information could still not be confirmed, it was marked as “not available”. Two reviewers independently extracted all variables from the included trials. Any disagreements were resolved through consensus with a third reviewer.

### 2.4 Data analysis

Categorical variables were presented as frequencies and percentages. Data were analyzed using SAS (version 9.4; SAS Institute, Cary, NC, USA). The temporal trend in the number of registered clinical trials was examined by applying a simple linear regression model. The model parameters (intercept and slope) were estimated, along with their corresponding 95% confidence intervals (95% CI). An “not available” category was used for missing data.

## 3 Results

### 3.1 Number of pediatric clinical trial registrations

**Figure 1:**
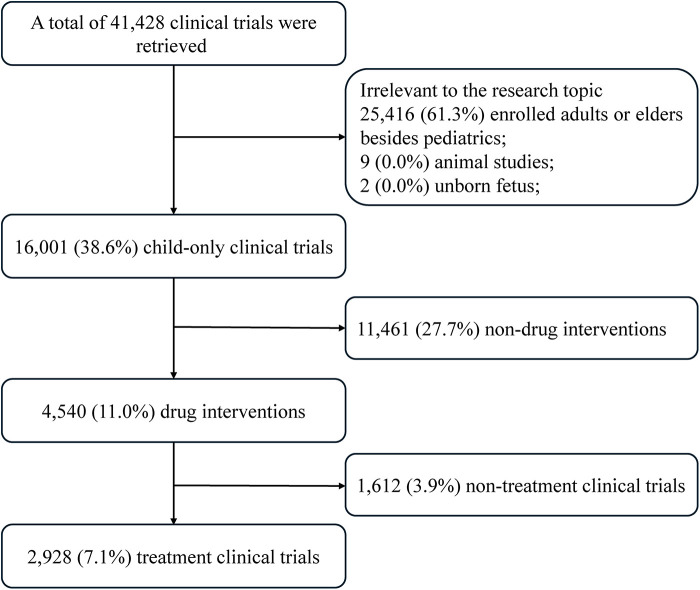
Flowchart of the study selection process for therapeutic drug intervention trials in pediatrics.

A total of 41,428 clinical trials were initially identified from the database. Of these trials, 25,416 (61.3%) were excluded because they enrolled adults or older participants in addition to pediatric populations. Additionally, nine animal studies and two trials involving unborn fetuses were removed. After these exclusions, 16,001(38.6%) clinical trials were confirmed as pediatric-only studies. Among these trials, 11,461(27.7%) involved nondrug interventions and were excluded to narrow the analysis to drug-related research. This process resulted in 4,540 (11.0%) drug intervention trials. From these trials, 1,612 (3.9%) trials were excluded because they were categorized as nontreatment clinical trials. The final dataset comprised 2,928 (7.1%) treatment clinical trials involving pharmacological interventions specifically targeted at pediatric populations (Figure 1).

### 3.2 Characterization of the included trials

Of the 2,928 eligible trials, most included both boys and girls (2,774, 94.7%) and enrolled only patients who were undergoing treatment (2,676, 91.4%). The majority had 100 or fewer participants (1,975, 67.5%). Among these small size trials, the numbers of Phase 1, Phase 1/Phase 2, Phase 2, Phase 2/Phase 3, Phase 3, and Phase 4 trials were 275 (13.9%), 175 (8.9%), 487 (24.7%), 99 (5.0%), 409 (20.7%), and 329 (16.7%), respectively. Furthermore, a considerable number of small-sample trials were concentrated in the following disease areas: nervous system diseases (206, 10.4%), endocrine, nutritional and metabolic diseases (202, 10.2%), mental, behavioral and neurodevelopmental disorders (198, 10.0%), digestive system diseases (146, 7.4%), and respiratory system diseases (138, 7.0%). Within these five areas, the top two most frequently studied indications were: Duchenne muscular dystrophy (54, 2.7%) and epilepsy (35, 1.8%); diabetes (17, 0.9%) and obesity (12, 0.6%); autism spectrum disorder (73, 3.7%) and attention deficit hyperactivity disorder (40, 2.0%); ulcerative colitis (15, 0.8%) and Crohn's disease (13, 0.7%); asthma (44, 2.2%) and pulmonary infection (43, 2.2%).

Many trials utilized randomized allocation (1,906, 65.1%), adopted parallel-group designs (1,857, 63.4%), and incorporated masking (1,479, 50.5%). A total of 718 trials (24.5%) were international multicenter studies. Of all eligible trials, 1,230 (42.0%) were completed; 602 (20.6%) were recruiting; and 352 (12.0%) were suspended, terminated, or withdrawn for various reasons. Among all included trials (2,928), 1,104 (37.7%) had disclosed their results; these were obtained from either the ClinicalTrials.gov registry or the scientific literature (Embase, MEDLINE, and Web of Science). The distribution of study phases was as follows (Table 1): 306 (10.5%) in Phase 1, 187 (6.4%) in Phase 1/Phase 2, 618 (21.1%) in Phase 2, 161 (5.5%) in Phase 2/Phase 3, 810 (27.7%) in Phase 3, and 542 (18.5%) in Phase 4.

**Table 1:** Characteristics and designs of pediatric trials registered on ClinicalTrials.gov, 2015–2024 (*N* = 2,928).

Category	Number of Trials	Percentage of Total
Study Status		
Active Not Recruiting	220	7.5%
Completed	1,230	42.0%
Enrolling By Invitation	24	0.8%
Not Yet Recruiting	154	5.3%
Recruiting	602	20.6%
Suspended	20	0.7%
Terminated	211	7.2%
Unknown	346	11.8%
Withdrawn	121	4.1%
Study Results^a^		
No	1,824	62.3%
Yes	1,104	37.7%
Sex		
All	2,774	94.7%
Female	22	0.8%
Male	131	4.5%
NA	1	0.0%
Age		
Neonates	205	7.0%
Infants	129	4.4%
Children	555	19.0%
Adolescents	169	5.8%
Neonates and Infants	165	5.6%
Infants and Children	322	11.0%
Children and Adolescents	912	31.1%
Neonates, Infants and Children	104	3.6%
Infants, Children and Adolescents	252	8.6%
All Pediatrics	115	3.9%
Accepts Healthy Volunteers		
No	2,676	91.4%
Yes	251	8.6%
NA	1	0.0%
Phases		
Phase1	306	10.5%
Phase1|phase2	187	6.4%
Phase2	618	21.1%
Phase2|phase3	161	5.5%
Phase3	810	27.7%
Phase4	542	18.5%
Not applicable	304	10.4%
Enrollment		
≤100	1,975	67.5%
101–200	474	16.2%
201–300	170	5.8%
301–400	116	4.0%
401–500	58	2.0%
501–800	64	2.2%
801–1,000	22	0.8%
>1,000	49	1.7%
Allocation		
Non-Randomized	278	9.5%
Randomized	1,906	65.1%
NA	744	25.4%
Intervention Model		
Crossover	110	3.8%
Factorial	23	0.8%
Parallel	1,857	63.4%
Sequential	141	4.8%
Single	793	27.1%
NA	4	0.1%
Masking		
Double	1,218	41.6%
Open Label	1,449	49.5%
Single	261	8.9%
Number of Locations		
1	1,355	46.3%
2–10	626	21.4%
11–50	527	18.0%
51–100	118	4.0%
>100	37	1.3%
NA	265	9.1%
Geographical Distribution		
Domestic	1,945	66.4%
International	718	24.5%
NA	265	9.1%

NA, not available.

a The results of included trials were identified through a dual approach: (1) systematic searches of electronic bibliographic databases (Embase, MEDLINE, and Web of Science), and (2) direct examination of the “Results” tab for each trial record on ClinicalTrials.gov.

Participant enrollment spanned the four pediatric age groups (neonates, infants, children, and adolescents), with trials frequently including multiple groups. As detailed in Table 1, the children group was the most commonly included (2,260 trials), followed by adolescents (1,448 trials), infants (1,087 trials), and neonates (589 trials). The distribution of the 2,928 trials across all possible age group combinations is provided in Table 1.

On the basis of differences in origin, structural complexity, and production methods, also referencing the drug classification systems used by the FDA, European Medicines Agency (EMA), Japan's Pharmaceuticals and Medical Devices Agency (PMDA), and China's National Medical Products Administration (NMPA), drug interventions were classified into three categories: chemical drugs, biological products, and natural or traditional medicines (16). Corresponding numbers of clinical trials were 2,106 (71.9%), 774 (26.4%), and 48 (1.6%), respectively. The classification criteria for drug categories were shown in Supplementary Material S1.

### 3.3 Trends of pediatric trials

From 2015 to 2024, the annual number of interventional pediatric clinical trials using drugs for treatment ranged from 257 to 339. The median number of registered trials per year was 291. The highest number was recorded in 2015 (*n* = 339 trials), whereas the lowest was observed in 2022 (*n* = 257 trials). The observed pattern suggested a gradual decline in the registration of pediatric treatment drug trials over the past decade. A linear regression analysis was performed. The intercept (95%CI) was 322.93 (289.5, 356.4), while the slope (95% CI) was −5.48 (−10.9, −0.09). The regression equation was as follows:

y=−5.48x+322.93

A coefficient of determination (R^2^) value of 0.41 indicated a moderate downward trend. On average, the number of pediatric treatment trials decreased by approximately 5.48 trials a year. The annual number of trials is presented in Supplementary Material S2.

### 3.4 Analysis of therapeutic areas

Among the 2,928 pediatric clinical trials analyzed, the top 10 therapeutic areas accounted for 78.7% of all trials, indicating a strong concentration of research efforts in a limited range of disease categories.

Studies on mental, behavioral, or neurodevelopmental disorders accounted for the largest proportion of trials (319, 10.9%), with 95 and 86 trials specifically investigating Autism Spectrum Disorder (ASD) and Attention Deficit Hyperactivity Disorder (ADHD), respectively. The remaining nine top areas were: anesthesia (310, 10.6%); neurologic disorders (273, 9.3%); endocrine, nutritional, or metabolic diseases (270, 9.2%); respiratory disorders (267, 9.1%); certain infectious or parasitic diseases (218, 7.4%); gastrointestinal disorders (214, 7.3%); developmental anomalies (167, 5.7%); neoplasms (134, 4.6%); and conditions originating in the perinatal period (132, 4.5%). These 10 disease areas represented a total of 2,304 trials (78.7%), indicating the concentration of research in specific pediatric disease categories. Furthermore, a cross-analysis was performed between therapeutic areas and trial phases; detailed information can be found in Supplementary Material S3. In the pivotal Phase 3 stage, the most active disease areas were endocrine, nutritional or metabolic diseases (101, 12.5%), respiratory system diseases (95, 11.7%), and mental, behavioral or neurodevelopmental disorders (87, 10.7%). Across the spectrum from Phase 1 to Phase 3, mental, behavioral or neurodevelopmental disorders emerged as a relatively mature field, with trials distributed evenly across stages and maintaining substantial presence in Phase 2 (11.0%), Phase 2/Phase 3 (8.7%), and Phase 3 (10.7%). In contrast, clinical trials for neoplasms (cancers) became less frequent in later phases, with 32 trials in Phase 1 (accounting for 10.5% of all Phase 1 trials) but only 13 in Phase 3 (accounting for just 1.6% of all Phase 3 trials).

### 3.5 Gender eligibility for enrollment

2,774 (94.7%) trials enrolled both male and female participants, while 153 trials (5.2%) restricted participation to either males or females. Cross-tabulation revealed differences in sex-specific enrollment across certain disease areas (Supplementary Material S4). Among trials that enrolled only females, 10 (45.5%) focused on developmental anomalies, specifically Rett syndrome and Turner syndrome. Among male-only trials, 68 (51.9%) were related to nervous system disorders, all evaluating treatments for Duchenne muscular dystrophy, and another 12 (9.2%) focused on diseases of the blood or blood-forming organs, all involving hemophilia treatment.

### 3.6 Pharmaceutical dosage form

The 2,928 included studies were categorized into 6 groups by dosage form: gaseous (e.g., aerosols, inhalations, and gases), liquid (e.g., solutions, syrups, elixirs, suspensions, emulsions, liposomes, and lotions), semisolid (e.g., ointments, creams, gels, pastes, and transdermal patches), solid (e.g., powders, tablets, capsules, and granules), other (defined as trials where the same drug had more than one physical dosage form falling into the above classes), and not available. The distribution was as follows: gaseous formulations (161, 5.5%), liquid formulations (1,562, 53.3%), semisolid formulations (92, 3.1%), solid formulations (703, 24.0%). We observed that in 52 trials (1.8%), the sponsor employed two or more dosage forms for the same drug to accommodate pediatric patients across different age groups. Among these, the physical dosage forms used in 40 trials (1.4%) could not be simply classified as either solid or liquid and thus fell into the “others” category as defined above. Detailed data were provided in Supplementary Material S5. Additionally, 370 studies (12.6%) did not report dosage form information and were therefore categorized as “not available.” Liquid and solid formulations were the most frequently studied dosage forms, together accounting for 77.3% of all clinical trials.

**Figure 2:**
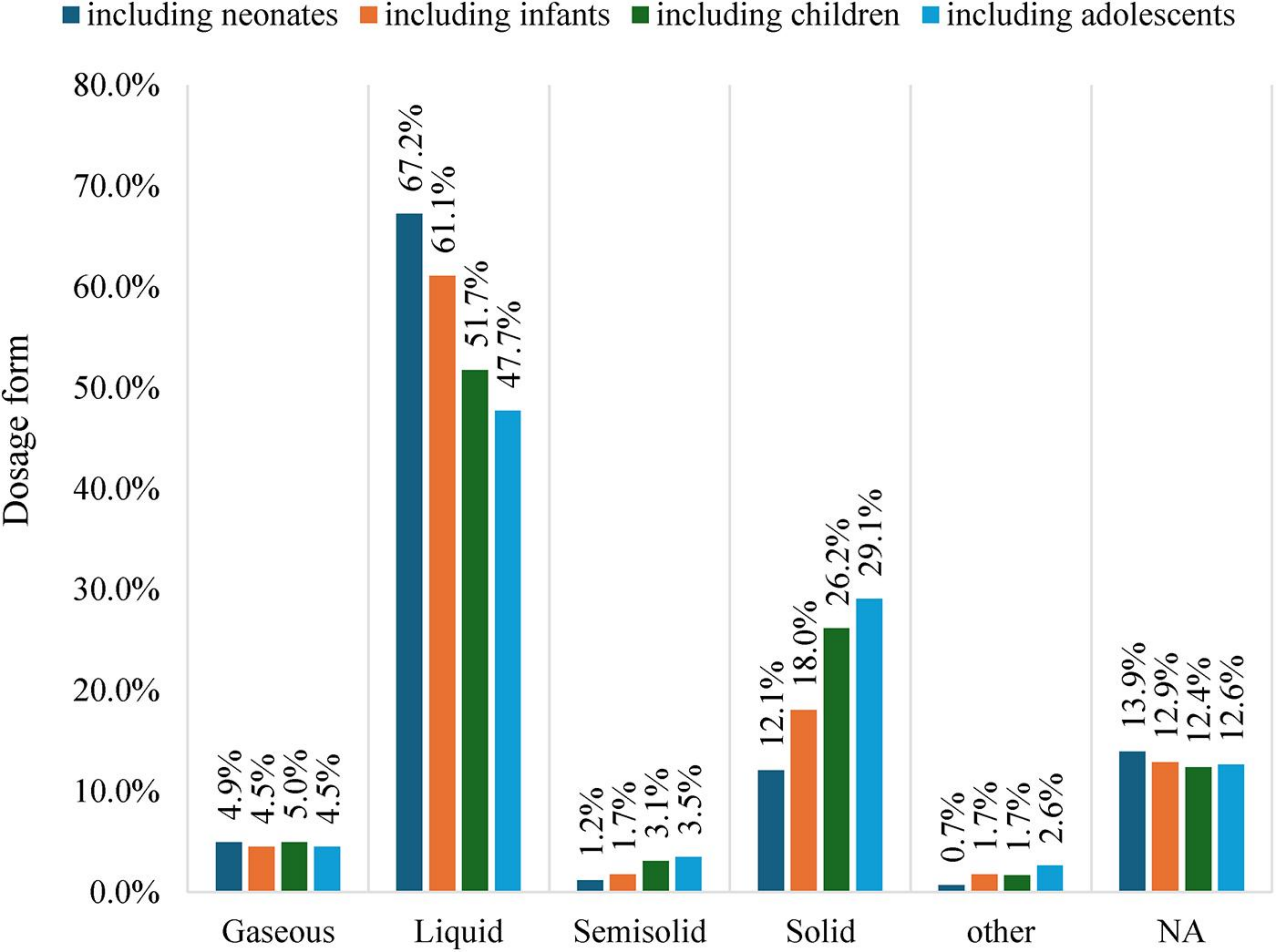
Distribution of dosage forms by age subgroup in the pediatric trials. Age subgroups are defined as follows: Neonates, Infants, Children, and Adolescents (see Methods). “Including neonates” refers to the enrolled patients' age range covering birth to 1 month; “including infants” covers 1 month to 2 years; “including children” covers 2–12 years; and “including adolescents” covers 12–17 years.

To assess dosage form preferences across pediatric subpopulations, we determined the distribution of each dosage form by age group. The preference for liquid formulations decreased with age, from 67.2% in neonates to 47.7% in adolescents. By contrast, the use of solid formulations exhibited an upward trend, increasing from 12.1% in neonates to 29.1% in adolescents. Although semisolid formulations exhibited an age-related pattern similar to that of solid formulations, their overall use remained low, resulting in less pronounced variations across age groups. The use of gaseous formulations remained relatively stable across the four age groups (Figure 2).

### 3.7 Administration route

On the basis of classifications outlined by the FDA and supported by the recent literature, the primary routes of drug administration in this study were grouped into seven categories: enteral, inhalation, local injection, local non-injection, parenteral, other (involving more than one route of administration), and not available. Among the 2,928 pediatric clinical trials analyzed, the distribution of administration routes was as follows: enteral, 1,143 trials (39.0%); inhalation, 113 trials (3.9%); local injection, 149 trials (5.1%); local non-injection, 296 trials (10.1%); parenteral, 1,041 trials (35.6%); other, 11 trials (0.4%), and not available, 175 trials (6.0%). The enteral and parenteral routes were the most common in the dataset.

**Figure 3:**
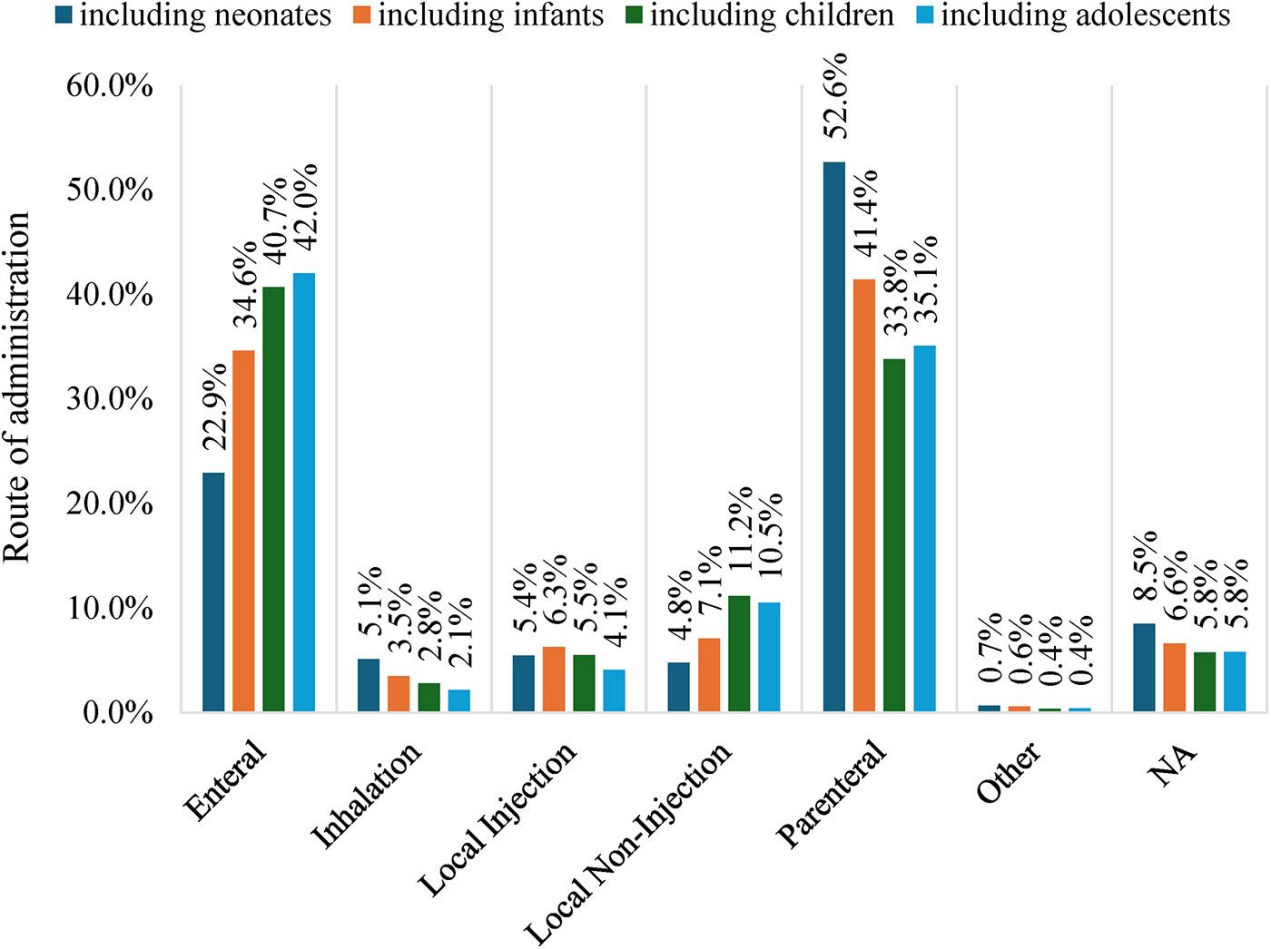
Distribution of drug administration routes by age subgroup in the pediatric trials. Age subgroups are defined as follows: Neonates, Infants, Children, and Adolescents (see Methods). “Including neonates” refers to the enrolled patients' age range covering birth to 1 month; “including infants” covers 1 month to 2 years; “including children” covers 2–12 years; and “including adolescents” covers 12–17 years.

Analysis by age group revealed that enteral and parenteral administration were the most frequently used routes across the four pediatric subgroups. The use of enteral administration increased with patient age, rising from 22.9% in the neonate group to 42.0% in the adolescent group. By contrast, parenteral administration was more common in neonates and infants than in older children and adolescents. Additionally, the use of the inhalation route gradually declined with increasing age, with the highest frequency in younger children and the lowest in adolescents (Figure 3).

## 4 Discussion

The Best Pharmaceuticals for Children Act (BPCA) and the Pediatric Research Equity Act (PREA) were aimed at promoting pediatric drug development through incentives and mandates, respectively. The 2012 FDA Safety and Innovation Act (FDASIA) made BPCA and PREA permanent and required all sponsors to submit an Initial Pediatric Study Plan (iPSP). This regulatory framework was designed to advance pediatric drug development through a proactive and flexible approach. The present study revealed that pediatric drug clinical research has not significantly expanded over the past decade; instead, it has declined with fluctuations. The number of pediatric clinical studies peaked in 2015 (339) and 2016 (328), potentially reflecting policy influences. However, the implementation of PREA inadvertently created a loophole enabling orphan drugs to avoid pediatric studies. The FDA moved to close this gap with a 2017 draft guidance (17). A slight increase in pediatric studies was observed in 2018 (300) compared to 2017 (287). The subsequent decrease in recent years may be partly attributable to the impact of the COVID-19 pandemic (18). The pandemic had a negative impact on non-COVID-19 clinical trial activities in the United States, Europe, and other regions (19, 20). On the other hand, pediatric drug development faces multiple challenges: First, regulatory challenges. Although ICH Harmonized Guideline E11 aims to provide a unified framework and basic principles for global pediatric drug clinical development, regulatory requirements across regions are often fragmented (21, 22). Second, technical challenges and ethical barriers. Evaluating drugs in children is more complex than in adults, owing to heightened safety concerns, recruitment difficulties, and the need for long-term follow-up (22). Finally, funding and priority challenges. Financial return is the key considerations for investors. Compared to adult-focused research and development, investment in pediatric research is limited. Several major pharmaceutical companies have closed their pediatric drug centers and focused on more profitable adult markets (22).

Zhong et al. (6) analyzed pediatric trials registered on ClinicalTrials.gov from 2008 to 2019, and found no growth in the number of pediatric drug trials during this period: 3,427 in 2008–2010, 3,066 in 2011–2013, 3,457 in 2014–2016, and 3,398 in 2017–2019. Moreover, the proportion of drug trials among all interventional pediatric studies showed a decreasing trend, dropping from 48.8% to 28.9%. Although Zhong's research differed from ours in terms of the scope of pediatric trials included, the observed trends consistently indicated limited growth in pediatric drug clinical research. Collectively, the overall situation of pediatric drug development has continued to face difficulties over the period from 2008 onward.

In order to promote pediatric drug development, international harmonization is of paramount importance. Building on existing collaborations, global regulatory authorities should strengthen cooperation to harmonize regulatory requirements and trial designs. The establishment of a single global pediatric development plan would be more efficient than navigating multiple disparate regional requirements. More specialized guidance, informed by the latest research, will help drug developers overcome technical challenges. For instance, the release and implementation of ICH E11A (Pediatric Extrapolation) in 2024 could provide significant support for pediatric drug development (23). Additionally, regulatory authorities should establish a more attractive framework for pediatric drug research by implementing new incentives. These could include granting exclusivity periods that are proportional to a study's complexity, cost, and public health value, moving beyond a one-size-fits-all reward. A notable precedent for this flexible approach is the European Commission's 2023 pharmaceutical legislation reform proposal, which introduced a variable-duration regulatory data protection system to replace the previous fixed 8-year period. This system directly links market exclusivity to a product's contribution to public health goals. Under it, the baseline data protection period is reduced, but companies can earn additional protection—up to a maximum of 12 years—by addressing unmet medical needs, ensuring supply security, and launching their products in all European Union member states in a timely manner (24).

Clinical trials provide reliable evidence of treatment outcomes through rigorously controlled testing on human participants. Pediatric trials, however, are more challenging to conduct than adult trials because of the unique physiological characteristics of children and certain ethical considerations (25). Clinical research involving children is further complicated by factors such as rare diseases, parental or guardian reluctance, and recruitment difficulties, which are key contributors to the failure of many pediatric trials (26). An appropriate sample size is crucial in clinical research (27). Of the analyzed trials, 1,975 (67.5%) enrolled no more than 100 participants. These trials included not only early exploratory studies but also 738 trials that were already in Phase 3 or Phase 4. The diseases studied were not limited to rare conditions; several involved common pediatric conditions such as respiratory tract infections, asthma, atopic dermatitis, childhood obesity, and ADHD. Pasquali et al. (5) analyzed pediatric clinical studies registered on ClinicalTrials.gov from 2005 to 2010 and identified a similar trend: 49.6% of pediatric trials enrolled fewer than 100 participants. They concluded that deriving clinically meaningful and generalizable information from such small-scale studies often presents substantial challenges. Developing clinical practice guidelines based on small sample trials was often questionable (28). Cao et al. (27) demonstrated that the results and conclusions, in terms of estimates of means, medians, Pearson correlations, chi-square test, and *P* values, are unreliable with small samples. In recent years, researchers have increasingly used the Bayesian statistical extrapolation method, which uses adult data to make inferences for pediatric populations, to address the aforementioned challenges (29). By incorporating prior information from historical research, Bayesian methods can improve estimation efficiency and have the potential to produce more reliable effect estimates even with limited sample sizes (30).

Califf et al. (31) reported variation in age distribution among therapeutic areas. Among the trials registered in 2007–2010 on ClinicalTrials.gov that allowed pediatric enrollment, those focusing on mental health accounted for the highest proportion. We found that studies on mental, behavioral or neurodevelopmental disorders also accounted for the largest proportion (319, 10.9%). ASD, a common neurodevelopmental condition, is categorized within this group. Data from the Autism and Developmental Disabilities Monitoring (ADDM) Network indicated a rising trend in the prevalence of ASD among children aged 8 years in the United States, which escalated from 1 in 150 in 2000 to 1 in 31 in 2022 (32). This growing prevalence highlights significant unmet therapeutic needs, driving continued momentum for clinical research in this area.

In our study, analysis of the association between areas and trial phases indicated a relatively mature developmental landscape, as later phases constituted a substantial proportion. This represented a promising development for pediatric patients, yet it called for cautious optimism. The transition from clinical investigation to real-world application remains highly uncertain. For instance, despite nearly 100 clinical trials for ASD, the successful translation of research findings into approved drugs remains limited. Risperidone and aripiprazole are the only two medications approved by the FDA for pediatric irritability associated with ASD (33). To date, no medication has been approved for the core symptoms of ASD (34).

Nearly all trials (94.7%) enrolled both sexes in our study, a result consistent with the 96.4% reported by Pasquali (5). The remaining 5.3% of trials had sex-specific enrollment criteria. For instance, studies of Rett syndrome and Turner syndrome recruited females only, whereas those for Duchenne muscular dystrophy and hemophilia exclusively enrolled males. These enrollment patterns closely reflected known differences in disease incidence between sexes. Rett syndrome is a rare genetic neurological disorder that predominantly affects girls, with an estimated prevalence of about 1 in 10,000 live female births (35). Turner syndrome is a sex chromosome disorder affecting only females (36). Both Duchenne muscular dystrophy and hemophilia have a significantly higher prevalence in males than in females (37, 38).

The selection of dosage form and administration route is a major step in drug development. Multiple factors influence this decision: for example, the physical and chemical properties of the active pharmaceutical ingredient, the type of disease, and the age of the patient (39). Age plays a key role because children in different developmental stages vary in their suitability for and acceptance of specific dosage forms and administration routes. Regarding drug formulation, liquid (1,562, 53.3%) and solid (703, 24.0%) formulations were the most commonly used forms. With increasing patient age, the use of liquid formulations decreased (from 67.2% to 47.7%), whereas the use of solid formulations increased (from 12.1% to 29.1%). Regarding administration route, enteral and parenteral were the most commonly used routes. The frequency of enteral administration increased with age (from 22.9% to 42.0%), whereas the preference for parenteral administration declined (from 52.6% to 33.8%). Older children are more likely to accept enteral administration because it is simpler, more convenient, and better tolerated. According to the matrix issued by EMA, oral liquids, such as solutions and suspensions, are more acceptable for younger children, whereas solid oral formulations are more acceptable for older children (40). This pattern reflects the practicality of oral liquids for younger children because these formulations allow for easier dosage adjustments and simpler administration (41). Swallowing oral solid medications, such as tablets and capsules, is generally more acceptable for older children than for younger ones (42). While these findings aligned with conventional expectations, several randomized crossover studies reviewed by Khan et al. (43) reported contrasting results. Studies showed that the acceptance of a mini-tablet, an innovative small flexible solid oral dosage form, was higher or equal to that of the oral liquid formulation (43). With continuous exploration and practice, emerging technologies are expected to mature and fundamentally transform the design of pediatric drug dosing regimens.

Medicines with age-appropriate administration routes and dosage forms can improve patient acceptance and compliance while ensuring greater dosing accuracy (44). We found that only 52 (1.8%) clinical trials explicitly specified in their registered protocols that different formulations would be used for children of different age groups, indicating that most studies did not design formulations tailored to the age of the enrolled participants. The development of age-specific formulations faced considerable challenges. Given that children are in a constant stage of growth, changes and differences in membrane permeability, enzymatic activities, pharmacokinetics, and pharmacodynamics lead to different responses of active substances in different age subgroups (39, 45). In addition, dosing flexibility, sensory properties (organoleptics), and safety must also be considered and carefully chosen during formulation development (46). As required by drug regulators such as the FDA, each new formulation requires its own regulatory approval, which requires separate research and development, stability testing, and safety assessments, adding significant time and cost to the drug development timeline (47). Collectively, these factors diminish the financial return on developing multiple pediatric formulations, making them unattractive to pharmaceutical companies. While the above observation could reflect a need for greater investment in formulation science, it must be interpreted with caution. Alternative explanations could include the demographic composition of our dataset, which may be skewed toward older pediatric populations where formulation differences were less critical, and the legitimate granting of regulatory waivers—a provision of both the PREA and the EU Pediatric Regulation (No 1901/2006), which exempts sponsors from developing formulations for specific age groups due to low disease prevalence.

Recent reviews on adverse drug reactions and pediatric risk factors have shown that age, comorbidities, polypharmacy, and hospitalization patterns shape adverse event profiles differently from those in adults (48, 49). There is an urgent need for pediatric-specific safety endpoints, age-stratified analyses, and adapted trial designs. Dosage form is a key consideration in clinical trial design. Future efforts should focus on developing suitable dosage forms that are customized for each pediatric age subgroup. Based on current formulation technology, minitablets and chewable tablets are practicable options for oral drug delivery (50–52). Buccal drug delivery also seems to be a good modality for pediatric-friendly dosage forms (39).

We noted instances of missing data and outdated information in the registry platform. Based on these findings, we recommend that Clinical trial registries (e.g., ClinicalTrials.gov) enhance data completeness and transparency. Regarding dosage form information, for instance, registry platforms could be improved by: (1) refining the “age” classification to include specific pediatric subgroups (neonates, infants, children, and adolescents), and (2) introducing a mandatory data field within the “arms and interventions” section to describe dosage forms and administration routes. This field would be automatically triggered for any study enrolling pediatric participants, thereby encouraging researchers to incorporate appropriate formulation design into their clinical protocols from the outset. In 2022, European Union Clinical Trials Information System (EU-CTIS) was launched by the EMA to serve as a unified public portal for clinical trial data in the EU and European Economic Area (EEA). This centralized platform is dedicated exclusively to the registration of interventional clinical trials for medicinal products and has the potential to improve trial efficiency and streamline regulatory processes. CTIS ensures comprehensive transparency through publicly accessible documents. For instance, it incorporates a dedicated field labeled “Age range secondary identifier” to specify the exact age range of participants, which allows users to quickly assess the age distribution of the enrolled population. Furthermore, sponsors are required to provide detailed product information—including the medicinal product's pharmaceutical form, route of administration, and maximum treatment duration —within the product-related sections. Additionally, the system maintains stringent criteria for deferral approvals. Collectively, these measures are expected to enhance the transparency of trial information and promote timely public disclosure (53).

A recent meta-analysis on off-label and unapproved use of pediatric medications revealed that 56% of pediatric prescriptions globally involve off-label use or medications without formal approval. This finding underscores the consequences of insufficient investment in pediatric drug development—a shortage of clinical trials and lack of age-appropriate formulations ultimately compromises clinical practice and patient safety (54). There is an urgent need for coordinated action by regulatory agencies and the pharmaceutical industry to address this critical issue.

We conducted a comprehensive longitudinal analysis of pediatric interventional drug clinical trials registered in ClinicalTrials.gov from 2015 to 2024, addressing a gap in the literature. By systematically reviewing all pediatric-only interventional drug trials, this study not only presented global trends in pediatric drug development but also reflected the evolution of pediatric clinical research under the current regulatory framework. A limited number of trials had designed age-appropriate formulations for different pediatric age groups. This finding primarily illuminated a landscape of limited age-specific formulation development, the underlying causes of which were likely multifactorial and warrant further investigation. Furthermore, our analysis of trial characteristics revealed an abundance of small-sample trials, including those in non-rare diseases. While robust traditional study design remains important, the effective use of advanced methodological and regulatory frameworks was critical to generating reliable evidence from the small-sample trials that characterize pediatric research (21, 29, 30, 55).

This study has some limitations. First, the analysis relied solely on the ClinicalTrials.gov registry. Although this database is the largest and most open clinical trial registry platform globally, containing information on numerous international multicenter trials, it does not capture pediatric research data from all countries and regions. Second, the identification of disease classifications, dosage forms, and administration routes was based on keyword extraction and manual categorization. Despite the use of standard definitions and multiple rounds of cross-validation, classification bias may persist when registration information is vague or incomplete. Third, the analysis was limited to data extracted from the clinical trial registry and subsequent scientific publications. While we conducted literature searches to augment the registry records, we did not contact trial investigators to obtain unreported data. This approach may have resulted in the omission of some unpublished outcomes or updated trial characteristics, potentially affecting the comprehensiveness of our dataset. Future research should integrate data from multiple international registries to better capture global pediatric research trends. Additionally, employing advanced natural language processing techniques could improve classification accuracy, while direct investigator outreach would help validate and enrich the data landscape.

## 5 Conclusion

Over the past decade, the number of pediatric drug clinical trials has declined. Most were small in size, and few studies used age-tailored formulations. Stronger incentives may be needed to encourage companies to conduct these trials, promote the submission of marketing authorization applications for pediatric-appropriate medications, and to advance the development of drugs with age-appropriate formulations and administration routes. The ongoing refinement of regulatory frameworks, optimization of trial registry systems, and development of novel methodologies are expected to facilitate the successful translation of pediatric drugs into clinical practice.

## Data Availability

The datasets presented in this study can be found in online repositories. The names of the repository/repositories and accession number(s) can be found below: https://clinicaltrials.gov/.
