# Supplementary material for: Characteristics of pediatric interventional drug trials registered between 2015 and 2024 on ClinicalTrials.gov
Source: Front Pediatr. 2025 Dec 10;13:1695990. doi: 10.3389/fped.2025.1695990 (PMC12727638; doi:10.3389/fped.2025.1695990)
Supplement: Supplementary file 2 [file Table2.docx]

Figure S2: Annual Number of Pediatric Interventional Drug Trials Registered on ClinicalTrials.gov in 2015-2024
