# Supplementary material for: Characteristics of pediatric interventional drug trials registered between 2015 and 2024 on ClinicalTrials.gov
Source: Front Pediatr. 2025 Dec 10;13:1695990. doi: 10.3389/fped.2025.1695990 (PMC12727638; doi:10.3389/fped.2025.1695990)
Supplement: Supplementary file 3 [file Table3.docx]

Table S3 Distribution of Therapeutic Areas Across Clinical Trial Phases: Pediatric Trials Registered on ClinicalTrials.gov, 2015-2024 (N = 2,928)

| Therapy Area | Phase1  (N=306) | Phase1\|phase2  (N=187) | Phase2  (N=618) | Phase2\|phase3  (N=161) | Phase3  (N=810) | Phase4  (N=542) | Not applicable  (N=304) | Total  (N=2,928) |
| --- | --- | --- | --- | --- | --- | --- | --- | --- |
|  | n(%) | n(%) | n(%) | n(%) | n(%) | n(%) | n(%) | n(%) |
| Mental, behavioural or neurodevelopmental disorders | 36(11.8) | 15(8.0) | 68(11.0) | 14(8.7) | 87(10.7) | 68(12.5) | 31(10.2) | 319(10.9) |
| Anesthesia | 21(6.9) | 8(4.3) | 32(5.2) | 21(13.0) | 44(5.4) | 105(19.4) | 79(26.0) | 310(10.6) |
| Diseases of the nervous system | 37(12.1) | 34(18.2) | 67(10.8) | 17(10.6) | 78(9.6) | 28(5.2) | 12(3.9) | 273(9.3) |
| Endocrine, nutritional or metabolic diseases | 16(5.2) | 33(17.6) | 55(8.9) | 12(7.5) | 101(12.5) | 41(7.6) | 12(3.9) | 270(9.2) |
| Diseases of the respiratory system | 18(5.9) | 2(1.1) | 47(7.6) | 16(9.9) | 95(11.7) | 61(11.3) | 28(9.2) | 267(9.1) |
| Certain infectious or parasitic diseases | 20(6.5) | 13(7.0) | 46(7.4) | 22(13.7) | 44(5.4) | 58(10.7) | 15(4.9) | 218(7.4) |
| Diseases of the digestive system | 25(8.2) | 11(5.9) | 37(6.0) | 8(5.0) | 58(7.2) | 40(7.4) | 35(11.5) | 214(7.3) |
| Developmental anomalies | 23(7.5) | 17(9.1) | 57(9.2) | 11(6.8) | 27(3.3) | 12(2.2) | 20(6.6) | 167(5.7) |
| Neoplasms | 32(10.5) | 16(8.6) | 44(7.1) | 6(3.7) | 13(1.6) | 14(2.6) | 9(3.0) | 134(4.6) |
| Certain conditions originating in the perinatal period | 21(6.9) | 8(4.3) | 30(4.9) | 6(3.7) | 34(4.2) | 21(3.9) | 12(3.9) | 132(4.5) |
| Diseases of the skin | 11(3.6) | 3(1.6) | 31(5.0) | 3(1.9) | 47(5.8) | 23(4.2) | 3(1.0) | 121(4.1) |
| Diseases of the blood or blood-forming organs | 7(2.3) | 3(1.6) | 26(4.2) | 6(3.7) | 43(5.3) | 19(3.5) | 6(2.0) | 110(3.8) |
| Diseases of the immune system | 8(2.6) | 9(4.8) | 19(3.1) | 1(0.6) | 31(3.8) | 11(2.0) | 6(2.0) | 85(2.9) |
| Diseases of the circulatory system | 12(3.9) | 5(2.7) | 18(2.9) | 9(5.6) | 17(2.1) | 9(1.7) | 9(3.0) | 79(2.7) |
| Diseases of the visual system | 4(1.3) | 5(2.7) | 14(2.3) | 3(1.9) | 25(3.1) | 9(1.7) | 10(3.3) | 70(2.4) |
| Diseases of the genitourinary system | 5(1.6) | 2(1.1) | 13(2.1) | 2(1.2) | 30(3.7) | 5(0.9) | 6(2.0) | 63(2.2) |
| Diseases of the musculoskeletal system or connective tissue | 3(1.0) | 2(1.1) | 4(0.6) | 2(1.2) | 17(2.1) | 3(0.6) | 4(1.3) | 35(1.2) |
| Sleep-wake disorders | 3(1.0) | 0(0.0) | 6(1.0) | 0(0.0) | 6(0.7) | 8(1.5) | 3(1.0) | 26(0.9) |
| Diseases of the ear or mastoid process | 1(0.3) | 1(0.5) | 3(0.5) | 1(0.6) | 8(1.0) | 3(0.6) | 1(0.3) | 18(0.6) |
| Injury, poisoning or certain other consequences of external causes | 1(0.3) | 0(0.0) | 1(0.2) | 1(0.6) | 2(0.2) | 3(0.6) | 1(0.3) | 9(0.3) |
| Symptoms, signs or clinical findings, not elsewhere classified | 2(0.7) | 0(0.0) | 0(0.0) | 0(0.0) | 2(0.2) | 1(0.2) | 2(0.7) | 7(0.2) |
| Pregnancy, childbirth or the puerperium | 0(0.0) | 0(0.0) | 0(0.0) | 0(0.0) | 1(0.1) | 0(0.0) | 0(0.0) | 1(0.03) |
