# Supplementary material for: Characteristics of pediatric interventional drug trials registered between 2015 and 2024 on ClinicalTrials.gov
Source: Front Pediatr. 2025 Dec 10;13:1695990. doi: 10.3389/fped.2025.1695990 (PMC12727638; doi:10.3389/fped.2025.1695990)
Supplement: Supplementary file 4 [file Table4.docx]

Table S4 Cross-Tabulation of Therapeutic Areas and Gender in Pediatric Trials Registered on ClinicalTrials.gov in 2015-2024 (N = 2,928)

| Therapy Area | All (Female or Male)  (N=2,774) | Female Only  (N=22) | Male Only  (N=131) | Not applicable  (N=1) | Total  (N=2,928) |
| --- | --- | --- | --- | --- | --- |
|  | n(%) | n(%) | n(%) | n(%) | n(%) |
| Mental, behavioural or neurodevelopmental disorders | 311(11.2) | 2(9.1) | 6(4.6) | 0(0.0) | 319(10.9) |
| Anesthesia | 295(10.6) | 0(0.0) | 15(11.5) | 0(0.0) | 310(10.6) |
| Diseases of the nervous system | 204(7.4) | 1(4.5) | 68(51.9) | 0(0.0) | 273(9.3) |
| Endocrine, nutritional or metabolic diseases | 254(9.2) | 5(22.7) | 11(8.4) | 0(0.0) | 270(9.2) |
| Diseases of the respiratory system | 266(9.6) | 0(0.0) | 0(0.0) | 1(100.0) | 267(9.1) |
| Certain infectious or parasitic diseases | 213(7.7) | 3(13.6) | 2(1.5) | 0(0.0) | 218(7.4) |
| Diseases of the digestive system | 212(7.6) | 0(0.0) | 2(1.5) | 0(0.0) | 214(7.3) |
| Developmental anomalies | 153(5.5) | 10(45.5) | 4(3.1) | 0(0.0) | 167(5.7) |
| Neoplasms | 133(4.8) | 0(0.0) | 1(0.8) | 0(0.0) | 134(4.6) |
| Certain conditions originating in the perinatal period | 132(4.8) | 0(0.0) | 0(0.0) | 0(0.0) | 132(4.5) |
| Diseases of the skin | 121(4.4) | 0(0.0) | 0(0.0) | 0(0.0) | 121(4.1) |
| Diseases of the blood or blood-forming organs | 98(3.5) | 0(0.0) | 12(9.2) | 0(0.0) | 110(3.8) |
| Diseases of the immune system | 81(2.9) | 0(0.0) | 4(3.1) | 0(0.0) | 85(2.9) |
| Diseases of the circulatory system | 79(2.8) | 0(0.0) | 0(0.0) | 0(0.0) | 79(2.7) |
| Diseases of the visual system | 70(2.5) | 0(0.0) | 0(0.0) | 0(0.0) | 70(2.4) |
| Diseases of the genitourinary system | 57(2.1) | 0(0.0) | 6(4.6) | 0(0.0) | 63(2.2) |
| Diseases of the musculoskeletal system or connective tissue | 35(1.3) | 0(0.0) | 0(0.0) | 0(0.0) | 35(1.2) |
| Sleep-wake disorders | 26(0.9) | 0(0.0) | 0(0.0) | 0(0.0) | 26(0.9) |
| Diseases of the ear or mastoid process | 18(0.6) | 0(0.0) | 0(0.0) | 0(0.0) | 18(0.6) |
| Injury, poisoning or certain other consequences of external causes | 9(0.3) | 0(0.0) | 0(0.0) | 0(0.0) | 9(0.3) |
| Symptoms, signs or clinical findings, not elsewhere classified | 7(0.3) | 0(0.0) | 0(0.0) | 0(0.0) | 7(0.2) |
| Pregnancy, childbirth or the puerperium | 0(0.0) | 1(4.5) | 0(0.0) | 0(0.0) | 1(0.03) |
