# Supplementary material for: Characteristics of pediatric interventional drug trials registered between 2015 and 2024 on ClinicalTrials.gov
Source: Front Pediatr. 2025 Dec 10;13:1695990. doi: 10.3389/fped.2025.1695990 (PMC12727638; doi:10.3389/fped.2025.1695990)
Supplement: Supplementary file 5 [file Table5.docx]

Table S5. Dosage Form Profile of 52 Trials Employing Age-specific Formulations (N = 52)

| **NCT Number** | **Physical Dosage Form** | **Categories of Dosage Form** |
| --- | --- | --- |
| NCT03774082 | Tablet, or injection | Other |
| NCT03682640 | Tablet, oral suspension | Other |
| NCT04516967 | Tablet, or sprinkle capsule | Solid |
| NCT02464969 | Tablet, or oral solution | Other |
| NCT03741101 | Tablet, or oral solution | Other |
| NCT06321601 | Tablet, or oral solution | Other |
| NCT04411901 | Drops, tablets | Other |
| NCT05609630 | Tablet, oral solution | Other |
| NCT04624230 | Tablet, oral solution | Other |
| NCT03000439 | Tablet, or solution | Other |
| NCT05261139 | Tablet, or powder | Solid |
| NCT03934372 | Tablet, or age-appropriate formulation | Other |
| NCT05003986 | Tablet, oral suspension | Other |
| NCT02605122 | Capsules, or suspension | Other |
| NCT02563496 | Tablet, dispersible tablet | Solid |
| NCT04553419 | Capsule, oral suspension | Other |
| NCT06332534 | Tablet, oral solution | Other |
| NCT02592434 | Tablet, or oral solution | Other |
| NCT03384121 | Capsule, or oral suspension | Other |
| NCT04731103 | Tablet, or oral solution | Other |
| NCT03201003 | Capsules, sachets | Solid |
| NCT02559817 | Oral solution, or capsule | Other |
| NCT04966234 | Tablet, oral suspension | Other |
| NCT02850406 | Capsule, or tablet, or powder for oral suspension | Solid |
| NCT03379506 | Tablet, or oral granule | Solid |
| NCT02486406 | Tablet (>=12) or minitablet (<12) | Solid |
| NCT04641975 | Tablet, oral suspension | Other |
| NCT03491215 | Tablet, or oral solution, or capsule dispersed in liquid | Other |
| NCT02556099 | Capsule, or oral liquid | Other |
| NCT03650452 | Tablet or mini-tablet | Solid |
| NCT02601937 | Tablet, or oral suspension | Other |
| NCT04375800 | Capsule or sachet or tablet | Solid |
| NCT05439616 | Capsules, oral solution | Other |
| NCT02559570 | Capsule or oral solution | Other |
| NCT05714085 | Tablet, oral solution | Other |
| NCT05782907 | Tablet, oral solution | Other |
| NCT04088409 | Oral suspension (<6 years of age) or tablet | Other |
| NCT03725007 | Tablet, oral solution | Other |
| NCT02798471 | Tablet, oral suspension | Other |
| NCT03485677 | Capsule, oral liquid | Other |
| NCT05319353 | Tablet, or powder for oral suspension | Solid |
| NCT04759833 | Tablet, oral solution | Other |
| NCT04806451 | Capsule, oral solution | Other |
| NCT03429400 | Tablet, or oral solution | Other |
| NCT03005483 | Capsule, or oral solution | Other |
| NCT02793011 | Capsule, oral liquid | Other |
| NCT04925479 | Mini-tablet, or tablet | Solid |
| NCT03816176 | Injection，capsule | Other |
| NCT04447989 | Injection, powder for oral suspension | Other |
| NCT06626503 | Injection, tablet | Other |
| NCT03453177 | Solution for intravenous infusion, oral suspension | Liquid |
| NCT04218851 | Injection, tablet | Other |
